# Evidence of scrapie transmission to sheep via goat milk

**DOI:** 10.1186/s12917-016-0807-4

**Published:** 2016-09-17

**Authors:** Timm Konold, Leigh Thorne, Hugh A. Simmons, Steve A. C. Hawkins, Marion M. Simmons, Lorenzo González

**Affiliations:** 1 Animal Sciences Unit, Animal and Plant Health Agency Weybridge, New Haw, Addlestone, Surrey, KT15 3NB UK; 2 Virology Department, Animal and Plant Health Agency Weybridge, New Haw, Addlestone, Surrey, KT15 3NB UK; 3 Pathology Department, Animal and Plant Health Agency Weybridge, New Haw, Addlestone, Surrey, KT15 3NB UK; 4 Pathology Department, Animal and Plant Health Agency Lasswade, Pentlands Science Park, Bush Loan, Penicuik, Midlothian, EH26 0PZ UK

**Keywords:** Transmissible spongiform encephalopathy, Scrapie, Goat, Sheep, Milk, Colostrum, Transmission, Protein misfolding cyclic amplification, Prion protein, Genotype

## Abstract

**Background:**

Previous studies confirmed that classical scrapie can be transmitted via milk in sheep. The current study aimed to investigate whether scrapie can also be transmitted via goat milk using in vivo (new-born lambs fed milk from scrapie-affected goats due to the unavailability of goat kids from guaranteed scrapie-free herds) and in vitro methods (serial protein misfolding cyclic amplification [sPMCA] on milk samples).

**Results:**

In an initial pilot study, new-born lambs of two different prion protein gene (*PRNP*) genotypes (six VRQ/VRQ and five ARQ/ARQ) were orally challenged with 5 g brain homogenate from two scrapie-affected goats to determine susceptibility of sheep to goat scrapie. All sheep challenged with goat scrapie brain became infected based on the immunohistochemical detection of disease-associated PrP (PrP^sc^) in lymphoid tissue, with an ARQ/ARQ sheep being the first to succumb. Subsequent feeding of milk to eight pairs of new-born ARQ/ARQ lambs, with each pair receiving milk from a different scrapie-affected goat, resulted in scrapie in the six pairs that received the largest volume of milk (38–87 litres per lamb), whereas two pairs fed 8–9 litres per lamb, and an environmental control group raised on sheep milk from healthy ewes, did not show evidence of infection when culled at up to 1882 days of age. Infection in those 12 milk recipients occurred regardless of the clinical status, PrP^sc^ distribution, caprine arthritis-encephalitis virus infection status and *PRNP* polymorphisms at codon 142 (II or IM) of the donor goats, but survival time was influenced by *PRNP* polymorphisms at codon 141. Serial PMCA applied to a total of 32 milk samples (four each from the eight donor goats collected throughout lactation) detected PrP^sc^ in one sample each from two goats.

**Conclusions:**

The scrapie agent was present in the milk from infected goats and was able to transmit to susceptible species even at early preclinical stage of infection, when PrP^sc^ was undetectable in the brain of the donor goats. Serial PMCA as a PrP^sc^ detection method to assess the risk of scrapie transmission via milk in goats proved inefficient compared to the bioassay.

**Electronic supplementary material:**

The online version of this article (doi:10.1186/s12917-016-0807-4) contains supplementary material, which is available to authorized users.

## Background

Scrapie is a transmissible spongiform encephalopathy (TSE) of sheep and goats, characterised by accumulation of disease-associated prion protein (PrP^sc^) in brain and lymphoid tissues. Although the ability of the scrapie agent to transmit vertically and horizontally in small ruminants has been known for years, the sources or vehicles of infection were poorly understood and only recently has progress been made to establish which secretions and excretions are infectious. Disease transmission has been demonstrated in lambs fed milk from scrapie-affected ewes [1–3]. Studies on the infectivity of milk from small ruminants, which is used for human consumption, have become more relevant since the demonstration of the zoonotic potential of bovine spongiform encephalopathy (BSE) and the identification of naturally occurring BSE in goats [4, 5]. The only previous infectivity study, in which two 3 month-old goats were intracerebrally inoculated with 1 ml of milk from an experimentally infected goat, failed to demonstrate transmission up to 29 months post inoculation [6]. At the time of that experiment, the influence of the prion protein genotype (*PRNP*) on scrapie susceptibility was unknown. It has now been established that susceptibility to scrapie in sheep is predominantly influenced by *PRNP* polymorphisms at codons 136 (alanine [A_136_] or valine [V_136_]), 154 (arginine [R_154_] or histidine [H_154_]) and 171 (glutamine [Q_171_] or R_171_), with ARQ/ARR and ARR/ARR being highly resistant against classical scrapie and VRQ/VRQ, ARQ/ARQ and VRQ/ARQ being highly susceptible [7]. More recent research has suggested that *PRNP* genetics in goats may also play a role on susceptibility to scrapie, with polymorphisms at codons 127 (serine [S_127_] instead of glycine [G_127_]), 142 (methionine [M_142_] instead of isoleucine [I_142_]), 146 (S_146_ and aspartic acid [D_146_] instead of asparagine [N_146_]), 154 (H_154_ instead of R_154_), 211 (Q_211_ instead of R_211_) and 222 (lysine [K_222_] instead of Q_222_) giving some protective effect against classical scrapie in goats [8].

Following our infectivity studies in sheep milk, the study reported here aimed to investigate whether scrapie from goats can be transmitted to sheep via milk by the oral route. Lambs were used as milk recipients because they could be sourced from a closed flock of known classical scrapie-free status. However, previous transmission studies had only confirmed the susceptibility of goats to sheep scrapie by natural or oral infection [9, 10] but not vice-versa. Therefore, a pilot study was initiated to determine whether sheep were susceptible to goat scrapie following oral challenge with brain homogenate, which would mimic the route for a further milk transmission experiment, and to assess which genotype, VRQ/VRQ or ARQ/ARQ, was more susceptible. This report summarises the final results of this pilot and the subsequent milk transmission study, preliminary results of which were reported previously [11]. The findings are compared with those of serial Protein Misfolding Cyclic Amplification (sPMCA) applied to goat milk, since this method had been able to detect PrP^sc^ in milk samples from scrapie-affected sheep, which also transmitted scrapie to lambs [3].

## Methods

An overview of the study is given in the Additional file 1 “schematic summary of the study”. The goats referred to in the text were from a herd with naturally occurring scrapie that was culled according to regulation No 999/2001 of the European parliament and of the council laying down rules for the prevention, control and eradication of certain transmissible spongiform encephalopathies, which provided the opportunity to study some of the animals and collect samples prior to cull.

### Pilot study to determine susceptibility of sheep to caprine scrapie

Brain homogenates were prepared from two Anglo-Nubian goats naturally infected with scrapie [12] according to established methods [13]: G1460 with I_142_I (subsequently named “BII”) and G1451 with I_142_M (subsequently named “BIM”) *PRNP* genotypes. More details on these goats, including clinical signs, are provided elsewhere [14].

Six Cheviot ewes with twin pregnancy were acquired from a classical scrapie-free flock [15], which produced eleven Cheviot lambs with *PRNP* genotypes VRQ/VRQ (*n* = 6) and ARQ/ARQ (*n* = 5); the sixth ARQ/ARQ lamb was culled shortly after birth due to injury. VRQ lambs were selected because of the high attack rate in sheep of this genotype fed milk from scrapie-affected sheep [3]. As it was also known for sheep that cross-*PRNP* genotype transmissions of scrapie resulted in considerably increased survival times [16], *ARQ* sheep were selected because it would match the *PRNP* genotype at these positions in goats, which lack polymorphisms at codons 136 and 171 [7]. *PRNP* genotyping was subsequently extended to other polymorphisms, particularly at codon 141 (leucine [L_141_] or phenylalanine [F_141_]), which has shown to influence disease in experimentally infected ARQ/ARQ sheep [16].

The lambs were orally challenged within 24 h after birth with 5 g (as 10 % w/v solution in physiological saline) of either BII or BIM brain homogenate. Lambs were raised by their dams until weaning at approximately 10 weeks of age and housed in three pens, two of which contained lambs challenged with either BII or BIM and one housed a mixture of lambs challenged with BII or with BIM (Table 1). Sheep were fed straw and concentrates after weaning, and lambs from the different pens were mixed from 35 months of age when the number of animals started to decline.

**Table 1:** Details of sheep orally challenged with caprine scrapie brain homogenate

Donor (inoculum)	Recipient^a^	*PRNP* codon 141	First ante-mortem detection of PrP^sc^ in RAMALT [days of age] (percentage of the survival time)	Survival time [days of age]
BIM	V1418	LL	648 (56 %)	1167
	V1419	LL	626 (51 %)	1220
	A1476^b^	FF	616 (42 %)	1464
	V1451	LL	1207 (83 %)	1452
	A1472	FF	641 (45 %)	1429
BII	V1424	LL	627 (54 %)	1154
	V1425	LL	627 (58 %)	1073
	A1473	LL	617^c^ (58 %)	1060
	A1474	LF	–––	269
	V1452	LL	625 (54 %)	1158
	A1471	FF	1201 (59 %)	2030

^a^V = VRQ/VRQ; A = ARQ/ARQ

^b^Female; all other sheep were castrated males

^c^Sheep with PrP^sc^ in palatine tonsil biopsy at 266 days of age

Scrapie infection status was determined by the immunohistochemical (IHC) examination of biopsies of recto-anal mucosa-associated lymphoid tissue (RAMALT) [3] done at 6 and 9 months of age, and of palatine tonsil [17] done at 9 months of age if the RAMALT result was negative, because this was the time point when breeding of milk recipient lambs with the correct genotype needed to be arranged. Further rectal biopsies at 20–21 months and 6-monthly thereafter were taken only from sheep with previous negative results.

Sheep were monitored twice daily by farm staff and were examined for neurological signs first at 20 months of age, then at 31 months and then usually monthly or more frequently depending on clinical status, using a short examination protocol for detection of scrapie [18]. A full neurological examination [13] was carried out prior to cull. Assessments were made blind without knowledge of the genotype or inoculum. The clinical end-point was reached when animals displayed progressive abnormalities in sensation (positive scratch test with or without alopecia, absent menace response) and movement (ataxia, limb weakness, tremor). The brain and a range of lymphoid tissues were taken from sheep that died or were culled at clinical end-point and scrapie was confirmed by IHC examination of formalin-fixed and wax-embedded samples of the obex and lymphoid tissues using rat monoclonal antibody R145 as described previously [19].

### Milk transmission study

Goats from the naturally infected herd [12] were transported to the APHA for milking prior to culling and necropsy. Milk was collected from eight animals that were subsequently confirmed scrapie-positive on IHC examinations for PrP^sc^. Post-mortem test examination of the goats included IHC examination of brain and lymphoid tissues (palatine tonsil, spleen, nictitating membrane, distal ileum, RAMALT, medial retropharyngeal, prescapular, prefemoral, distal jejunal and mammary lymph nodes as well as mammary glands with antibody R145 [20]. From day 6 (day 4 in goat G1415) of milk collection, a milk sample was tested weekly for somatic cell count by fossomatic counter at the National Milk Records plc, Chippenham, UK. The milk donors included the two goats that provided the brain for the pilot study. At the time of milk collection, the goats were of different scrapie status, preclinical or clinical, and at various stages of lactation; the exact lactation day was not known except for two goats, which also provided colostrum. Basic information about the donor goats is given in Table 2 (see [14] for more information on the clinical presentation). The colostrum and milk were stored at -80 °C for approximately 2 years and defrosted prior to being fed to lambs or subjected to in vitro PrP^sc^ detection (see below).

**Table 2:** Details of scrapie-affected goats that provided milk and/or had milk tested for PrP^sc^

Donor	Genotype codon 142	Clinical status	Post-mortem TSE status (LRS tissue & brain)^b^	CAEV status	PrP^sc^ in mammary gland	PMCA positive result in milk/ number of tests (days of lactation)	Weekly somatic cell count in milk [×10^3^ cells/ ml] (median)
Milk transmission study							
G1472	II	–	2/10 & N	–	absent	0/2 (1, 10, 19, 29)	1013, 1922, 993, 1514 (1003)
BII^a^	II	+	7/10 & P	–	absent	0/2 (5, 35); 1/3 (20), 0/3 (51)	878, 3707, 1152, 949, 3469, 1192 (1172)
G1143	II	±	9/10 & N	+	present	0/2 (1, 16, 30, 45)	86, 155, 163, 97, 262, 136 (145.5)
G1427^a^	II	–	10/10 & N	+	present	0/2 (6, 22, 47, 66)	956, 2445, 640, 439, 171, 415, 419, 219, 388, 551 (429)
G1465	IM	±	1/10 & N	+	absent	0/2 (1, 10, 18, 29)	413, 990, 2140, 978 (984)
G1415	IM	±	8/10 & P	–	absent	0/2 (1, 20, 39, 59)	1591, 279, 405, 439, 336, 431, 627, 369 (418)
G1383	IM	±	9/10 & P	–	absent	0/2 (1, 21, 42, 61)	4708, 312, 269, 219, 132, 148, 92, 107 (183.5)
BIM	IM	±	10/10 & P	–	absent	0/2 (1, 21); 1/3 (43); 0/3 (61)	2556, 271, 459, 6133, 267, 84, 347, 225 (309)
PrP^sc^ detection only by sPMCA							
G1376	II	–	0/10 & N	–	absent	0/2 (1, 10, 29); 0/3 (18)	853, 3420, 205, 3109 (1981)
G1136	IM	–	0/10 & N	–	absent	0/2 (1, 9, 18, 29)	354, 565, 378, 531 (454.5)
G1382	IM	–	0/10 & N	+	absent	0/2 (1, 19); 0/3 (10, 29)	135, 4388, 3835, 7007 (4112)
G1454	IM	±	0/10 & N	–	absent	0/2 (1, 10, 19, 29)	11, 8373, 469, 2808 (1639)
G1122	MM	–	0/10 & N	–	absent	0/2 (1, 37, 55); 0/3 (18)	238, 3646, 3586, >10000, >10000, 244, 215, 331 (1959)
G1115	MM	±	0/10 & N	–	absent	0/2 (1, 19, 37, 56)	48, 325, 535, 322, 300, 108, 198, 340 (311)

^a^also provided colostrum

^b^Post-mortem TSE status was determined by immunohistochemistry as described previously [12] and is shown as number of positive LRS tissues/number examined & PrP^sc^ in brain (P = positive; N = negative). CAEV status was determined serologically

– = clinically unremarkable or negative serology result; ± = inconclusive signs with regards to scrapie; + = clinically affected or positive serology result

Sixteen Cheviot lambs, born from ewes from the classical scrapie-free flock [15] that carried twin or single lambs, were fed colostrum from their dams for the first day and then switched to goat milk feeding. Goat milk was fed in the same order it was collected, i.e. milk collected at day 1 was fed first. These milk recipients were pairs of ARQ/ARQ lambs and the milk from each doe was split so that the lambs in each pair were fed approximately equal volumes of milk. Once all the goat milk was consumed, lambs were fed milk replacer (Lamlac, Volac International Ltd., Royston, UK), followed by a diet of straw and concentrates from a weaning age of approximately 10 weeks.

Five new-born lambs from the same classical scrapie-free flock, which were housed in the same building and shared the same air space as the others but kept in a separate pen, were maintained as environmental controls. These were kept with their dams until weaning and then fed the same diet as the goat milk-fed lambs. Each pen had a separate entrance and equipment; when equipment had to be shared (e.g. a weighing crate) it was decontaminated through exposure to 2 % hypochlorite solution (Haychlor Industrial bleach, Brenntag, Leeds, UK, diluted to appropriate concentration) for 1 h.

A blood sample was taken from all animals at 4–5, 6–7 months of age and prior to cull to test for antibodies against small ruminant lentivirus infection by Agar Gel Immunodiffusion Test (AGIDT) using the Maeditect test kit (APHA Weybridge) [21] as in previous milk transmission studies. Scrapie infection was monitored as described above for the pilot study: RAMALT sampling first at 9–10 months of age, then at 19 months and 6-monthly thereafter until first detection of PrP^sc^. The different pairs of milk recipients were kept in separate pens until scrapie infection was confirmed by RAMALT biopsy in at least one sheep within those pairs, at which time they were mixed.

Similar to the pilot study, the first clinical neurological assessments were conducted at approximately 24 months of age, then at 33 months and then monthly and at culling at clinical end-point, or due to intercurrent diseases. Assessments were made blind with regards to donor goat details, with the obvious exception of the environmental control group and later in the experiments also of the two pairs that had been fed the lowest volume, simply because these could not be mixed with the others. Post-mortem confirmation of scrapie was performed in the same way as in the pilot study.

Sheep in the pilot and milk transmission studies were kept up to clinical end-point to determine the clinical, pathological and molecular phenotype of caprine scrapie transmitted to sheep, which will be reported separately.

### PrP^sc^ detection by sPMCA

A brain homogenate from sheep from the same scrapie-free flock that provided the experimental animals [15] was selected as cellular prion protein (PrP^c^) substrate to mimic the in vivo transmission experiments where lambs were used as recipients. Firstly, amplification of PrP^sc^ from the caudal medulla of the two scrapie-infected goats used in the pilot experiment (BII and BIM) was tested using PMCA substrates prepared from sheep with *PRNP* genotypes VRQ/VRQ, ARQ/VRQ and ARQ/ARQ to determine the most suitable genotype. The methods for sample preparation, amplification and visualisation were described previously [22, 23]. Briefly, the seed and substrate brains were prepared by washing and liquidising in amplification buffer, and subsequently homogenised to provide a 10 % (w/v) homogenate.

Frozen whole milk samples (10 ml) were defrosted, clarified by centrifugation and the supernatants below the fat layer diluted 1:10 in the most suitable ovine PMCA substrate established previously (see above), supplemented with polyadenylic acid (P9403 Sigma-Aldrich) to increase the efficiency of amplification. Diluted samples were subjected to four rounds of sPMCA, with each round comprising 48 consecutive cycles of sonication and incubation. PMCA products were stored frozen at −20 °C until analysed by enzyme immunoassay (IDEXX HerdChek BSE-Scrapie Antigen Test Kit, IDEXX Laboratories, Westbrook, USA) using a modified protocol as described previously [23]. In each experiment negative control samples, PMCA substrate only and PMCA substrate spiked with a previously tested and known negative goat milk extract, were included to monitor both *de novo* synthesis and putative contamination. Following sPMCA all samples with an absorbance of 2 or more were considered positive. Milk samples were tested twice or three times in cases of a high negative or inconclusive result in one sample that warranted an additional assay to verify the result.

The milk from 14 goats was analysed, including the eight milk donors and another six that originated from the same herd but had tested negative for scrapie by laboratory tests (see Table 2 and [14] for more details about the animals). From each goat four samples were tested, which had been collected at equal intervals throughout the lactation. Samples were tested blind.

## Results

### Pilot study to determine susceptibility of sheep to caprine scrapie

Survival times, and the time of first detection of PrP^sc^ in RAMALT biopsies are listed in Table 1. Sheep A1473 was the one with the earliest detectable PrP^sc^ in palatine tonsil (266 days of age), which is why the ARQ/ARQ genotype was selected for milk recipients in the subsequent milk transmission study (see below). All sheep orally dosed with goat brain developed clinical signs of scrapie and this was confirmed by IHC examination of the brain. The only exception was sheep A1474 that died of an undiagnosed condition at 269 days of age and showed PrP^sc^ accumulation in the mesenteric lymph node.

The age at first detection of PrP^sc^ in RAMALT was 744 ± 243 days (mean ± standard deviation), that is at 56 ± 11 % of the survival time, which was 1321 ± 292 days of age. The outlier was sheep V1451, where PrP^sc^ in RAMALT was first detected at 83 % of the survival time, almost twice as long as the other sheep of the same genotype challenged with the same inoculum. Sheep A1471 did not show PrP^sc^ in RAMALT biopsies until 1201 days old but its survival time was also considerably longer than all other sheep. The shortest survival time was that of the single ALRQ/ALRQ sheep. AFRQ/AFRQ sheep had longer survival times than VRQ/VRQ sheep (167 days longer for BIM challenge and 902 days longer for BII challenge when mean survival times were compared). No comparison by statistical methods was carried out due to the small number of sheep per group.

### Milk transmission study

None of the sheep tested positive for antibodies against small ruminant lentiviruses at any of the two selected time points.

**Fig. 1:**
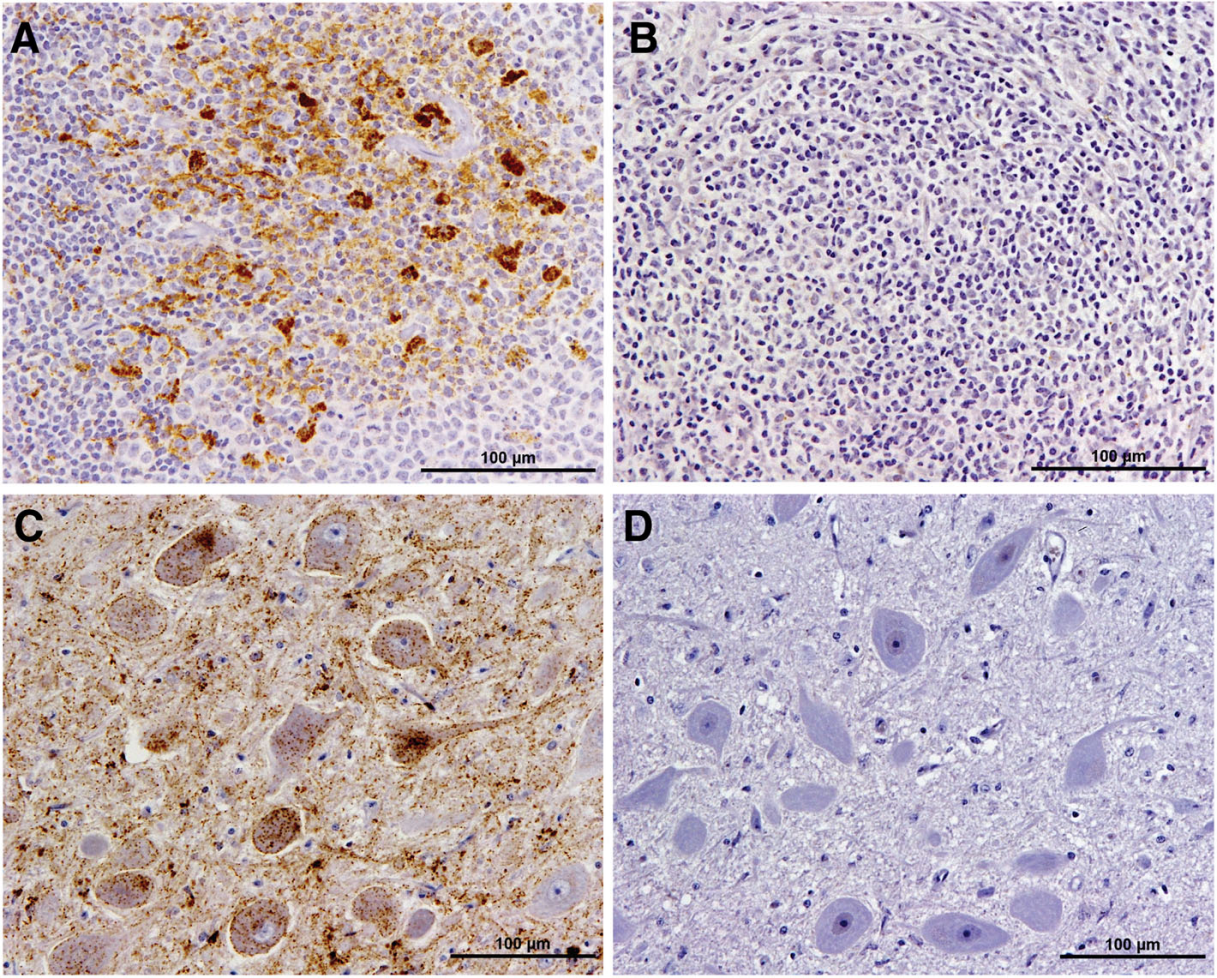
PrP^sc^ accumulation in brain and lymphoid tissue of goat scrapie milk recipient 1302 and comparison with control 1325. **a** RAMALT of sheep 1302 at 800 days of age; **b** RAMALT of sheep 1325 at 790 days of age. PrP^sc^ is visible in lymphoid follicles in goat scrapie milk recipient 1302 but absent in control sheep 1325. **c** Obex of sheep 1302 at 1466 days of age when culled; **d** Obex of sheep 1325 at 1867 days of age when culled. There is PrP^sc^ immunolabelling in the dorsal motor (parasympathetic) nucleus of the vagus nerve in goat scrapie milk recipient 1302 but no immunolabelling in control sheep 1325. Antibody R145

Survival times and time of first detection of PrP^sc^ in RAMALT (Fig. 1a) of the 16 sheep fed different volumes of milk from scrapie-infected goats are displayed in Table 3. None of the controls kept in the same building developed signs of scrapie and PrP^sc^ was not detected in either lymphoid tissues or the brain (Fig. 1b and d) of these animals.

**Table 3:** Details of sheep fed milk from scrapie-affected goats

Donor	Milk volume fed [litre]	Recipient	*PRNP* codon 141	First ante-mortem detection of PrP^sc^ (percentage of the survival time) or last negative rectal biopsy	Survival time [days of age]
G1383	87	1286^a^	LL	803 (62 %)	1285
	87	1287^a^	LF	585 (44 %)	1325
G1415	82	1319^a^	LL	1065 (95 %)	1122
	82	1320^a^	LF	795 (51 %)	1552
G1427	76^b^	1294^a^	LL	1045 (84 %)	1243
	76^b^	1293	LL	583 (45 %)	1284
BIM	63	1329	LL	1034 (80 %)	1298
	67	1302	LF	800 (55 %)	1466
G1143	57	1324^a^	LF	1366 (94 %)	1460
	57	1323^a^	LF	983 (66 %)	1486
BII	38^b^	1321^a^	LF	794 (60 %)	1333
	38^b^	1322	LF	1064 (71 %)	1493
G1465	8	1333^a^	LL	1358	1433
	9	1332^a^	LF	1761	1872
G1472	8	1330	LL	1361	1519
	8	1331	LF	1761	1872
Control	––––	1310	LF	1771	1882
Control	––––	1318	LF	1768	1880
Control	––––	1325	LF	1762	1867
Control	*––––*	1334	LF	*1758*	1864
Control	*––––*	1338	LL	*1752*	1858

^a^Female; all other sheep were castrated males; ^b^Included colostrum. Lambs were fed goat milk between 16 and 32 h (mean 24 h) after being born

All six sheep pairs fed relatively large volumes of goat milk (38–87 litres) were culled with clinical signs of scrapie and this was confirmed by IHC examination of the obex (Fig. 1c), while none of the two sheep pairs fed a small volume (8–9 litres) developed signs of scrapie or showed PrP^sc^ accumulation in any of the tissues examined. One in each scrapie-negative pair was lost due to intercurrent diseases (one found dead without any obvious cause, the other sheep had tetanus-like signs) at a time when other milk-fed sheep had already been culled with signs of scrapie. These four scrapie-negative lambs had been fed milk from goats with PrP^sc^ restricted to one or two lymphoid tissues and without PrP^sc^ in brain, while all 12 scrapie-infected recipients were fed milk from goats that had PrP^sc^ in seven or more lymphoid tissues examined and four of them also had PrP^sc^ in brain (see Table 2). Only two of the eight goats had detectable PrP^sc^ in the mammary gland, of which both transmitted scrapie to lambs.

For the three scrapie-infected milk recipient pairs that included one L_141_L and one L_141_F sheep, the survival times were 40 (G1383 donor), 168 (BIM donor) and 430 (G1552 donor) days longer in L_141_F than L_141_L sheep (see Table 3).

### PrP^sc^ detection by sPMCA

Amplification was not consistently achieved for goat brain samples using ovine PMCA substrates: BII amplified in VRQ/VRQ and ARQ/ARQ sheep substrate but not in ARQ/VRQ substrate, BIM only amplified in the VRQ/VRQ substrate. Therefore, ovine substrate of this last genotype was used to analyse the milk samples.

Of the six goats that transmitted scrapie to lambs via milk, two (BII and BIM) amplified PrP^sc^ in one of four tested samples each (and only in one third of the aliquots tested), whereas PrP^sc^ was not amplified in any of the others. Milk samples from the goats that were scrapie-negative by IHC examination of brain and lymphoid tissues were also negative in the PMCA assay.

## Discussion

Recent studies on scrapie transmission in small ruminants, particularly sheep, have widened our knowledge about sources of infectivity that could contribute to transmission from an infected dam to its offspring. A significantly increased incidence of scrapie has been found in the offspring of scrapie affected ewes, which is either the result of “in utero” or post-natal ewe to lamb transmission [24, 25]. Placenta is believed to be the main source of post-natal scrapie infection [26–28], contributing to infection most likely by horizontal transmission [29], while it has now been established through in vivo experiments that colostrum and milk are very effective sources for vertical scrapie transmission from dam to lamb [1–3, 30]. The study reported here addressed the question of whether milk from scrapie-affected goats is similarly infectious, and confirmed that milk from goats also harbours the scrapie agent and can transmit it to new-born lambs. The use of sheep lambs in this experiment was justified to guarantee that the recipients were scrapie-free, something that could have not been achieved with goat kids.

At the time the study was designed, no published information on the transmissibility of goat scrapie to sheep was available, and a pilot study was initiated to test the susceptibility of sheep to caprine scrapie. Oral challenge of new-born sheep with brain homogenate from two scrapie-affected goats produced a 100 % attack rate in both VRQ/VRQ and ARQ/ARQ sheep, while goat to sheep transmission with milk from infected goats was achieved in 75 % of the lambs dosed. A possible explanation for this difference is a potentially lower infectious titre in milk compared to brain combined with the effect of the species barrier. However, the lack of transmission observed in four sheep recipients might also have been due to the low volume of milk fed to them (8–9 litres) or to the donor goats being in an early stage of the incubation period, as shown by the restricted tissue distribution of PrP^sc^, or to both, but it does not exclude the possibility that such milk could have transmitted to goat kids. This hypothesis is supported by the observation that oral challenge with cotyledon homogenate from a scrapie-affected goat to new-born small ruminants produced scrapie in four of four goats but only in two of four VRQ/VRQ sheep [31]. Therefore, considering all these arguments and the fact that transmission of scrapie via milk was achieved even from goats in the preclinical phase, without detectable PrP^sc^ in the brain and without PrP^sc^ in the mammary gland, goat milk appears to be an efficient source for scrapie transmission.

Co-infection of sheep with scrapie and Maedi-Visna virus (MVV) has been associated with increased shedding of prions in milk [2, 30, 32]. In our experiment transmission of scrapie occurred independently of infection with caprine arthritis-encephalitis virus (CAEV, the caprine counterpart of MVV) in the donor goats and of the indurative mastitis associated with it [33] and none of the milk recipient lambs had serological evidence of small ruminant lentivirus infection. None of the goats exhibited clinical mastitis and the median somatic cell count in milk was not lower in goats that did not transmit scrapie compared to those that transmitted. It is known that there are multiple non-pathological factors, such as stage and number of lactation, and stress or change in diet, which affect somatic cell count and cause considerable variation even in milk from healthy udders of small ruminants [34]. This is therefore unreliable as specific indicator for TSE risk or as an indicator of udder health in goats [35].

Although the M_142_ allele of the caprine *PRNP* was associated with increased resistance to classical scrapie in the herd that provided the milk donors [12], milk from I_142_M goats was infectious. These goats showed a wide dissemination of PrP^sc^ in lymphoid tissues, which may correlate with increased presence of the agent in the blood and increased probability of shedding via milk. Indeed, this might be the case since the two goats that did not transmit scrapie had minimal involvement of the lymphoreticular system (LRS).

Any interpretation with regards to infectious titre of the scrapie agent by comparing survival times, which are used in mice as a crude method to estimate the infectious titre [36], is impossible due to the different milk volumes fed to pairs of lambs and their different genotypes. For example, feeding milk and colostrum from a clinically affected goat with PrP^sc^ in the brain (G1460, I_142_I) and milk from a goat that only had detectable PrP^sc^ in lymphoid tissue (G1143, also I_142_I) produced similar survival times in L_141_F milk recipients, which may suggest that the infectious titre was similar, but the latter goat produced almost twice as much milk so the volumes ingested by the lambs were different.

The poor correlation of the sPMCA results (PrP^sc^ detected in only two single samples from two goats) with the actual transmission results (successful transmission from six goats) was unexpected, given that PrP^sc^ was previously detected by sPMCA in similar volumes of milk samples collected from scrapie-affected sheep, which proved to transmit scrapie to lambs [3]. Although one recent study suggested that the levels of infectivity correlated well with detection of PrP^sc^ in tissues by laboratory tests, including PMCA, which was the most sensitive test [37], an earlier study indicated that the association between infectivity and laboratory test results was poor for the TSE sources and host *PRNP* genotypes used in the study [38]. The sPMCA results obtained from the two tested goat brains demonstrated that amplification was indeed affected by *PRNP* polymorphisms of the substrate source and/ or the goat brain donor and the choice of an ovine substrate may have contributed to the low amplification efficiency of goat scrapie, even though it transmitted to sheep in vivo. The quantity of PrP^sc^ in individual milk samples is likely to be so small that it is at the limit of detection with sPMCA, particularly in goats at an earlier stage of disease. The latter hypothesis is supported by the observation that both goats with the sPMCA-positive result in a single milk sample had a higher magnitude of PrP^sc^ accumulation in the brain than the others [20] and one displayed clear neurological signs of scrapie. We did not carry out a dilution study on brain to evaluate the limit of detection and also did not trial brain homogenate from ovinized transgenic mice (tg338) as substrate, which was used by other researchers and published after we had carried out our sPMCA experiments [39]. Tested milk samples were collected at different time points so it is unlikely that the failure to detect PrP^sc^ is due to inconsistent shedding of PrP^sc^ via milk. Amplification of PrP^sc^ by sPMCA may possibly also be affected by the peculiar nature of goat milk, which contains many cytoplasmic particles as a result of apocrine milk secretion [34] or any other, unknown inhibitors in goat milk.

The current study provided further evidence for the important effect of polymorphisms at codon 141 of the ovine *PRNP* on survival time. It has been previously shown that homologous oral scrapie transmission of scrapie in ARQ/ARQ sheep results in survival times that are approximately twice as long in sheep with the LF polymorphism compared to LL sheep [16]. A similar effect was seen in the current study where LL sheep had consistently shorter survival times than LF sheep fed milk from the same animal (40, 168 and 430 days respectively) although the difference in survival time was not as large as in the previously published study, which may be due to the species-barrier (sheep scrapie to sheep transmission [16] versus goat scrapie to sheep transmission here). However, an FF sheep survived for almost twice as long as an LL sheep although this was only established for the BII brain recipients which had this genotype combination. Although ARQ/ARQ sheep homozygous F_141_ are naturally susceptible to classical scrapie [40], this polymorphism is usually associated with higher susceptibility to atypical scrapie compared to L_141_ [41].

## Conclusions

This study confirmed that the classical scrapie agent is present in milk from scrapie-affected goats even at the pre-clinical stage when there is an absence of detectable PrP^sc^ in brain, and is able to transmit scrapie to susceptible species, although transmission was not achieved in milk volumes of less than 10 litres from goats with minimal lymphoid tissue involvement. Serial PMCA as a PrP^sc^ detection method to assess the potential risk of scrapie transmission via milk in goats was inferior to the in vivo transmission study.

## Additional file

Additional file 1: Schematic summary of the study. This file provides a graphical overview of the design of the pilot and milk transmission study and the overall results. (PDF 301 kb)
