# Supplementary material for: Evidence of scrapie transmission to sheep via goat milk
Source: BMC Vet Res. 2016 Sep 17;12:208. doi: 10.1186/s12917-016-0807-4 (PMC5027119; doi:10.1186/s12917-016-0807-4)

Pilot study

Does goat scrapie transmit to sheep?

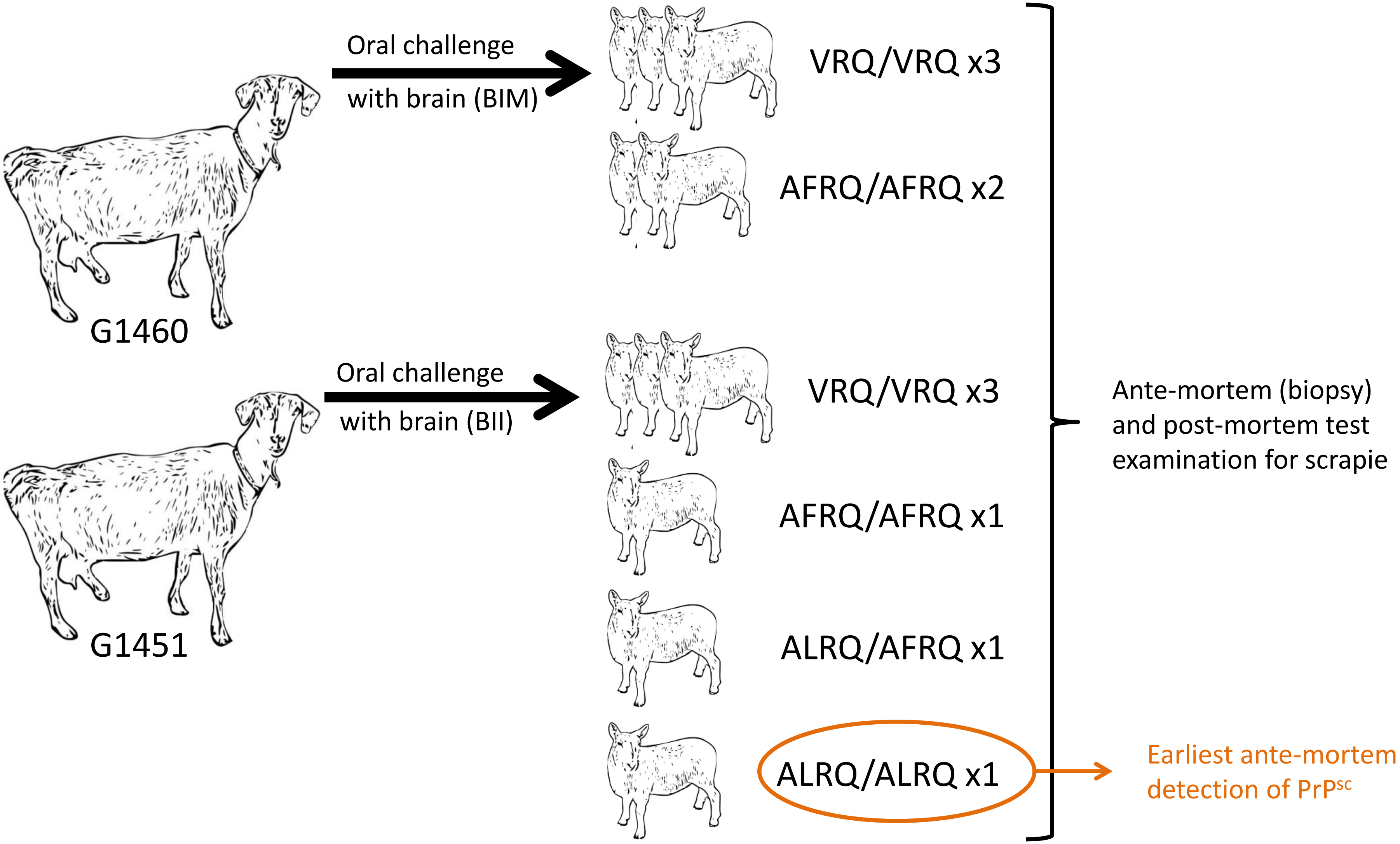

Milk study

Does milk from scrapie goats transmit the scrapie agent?

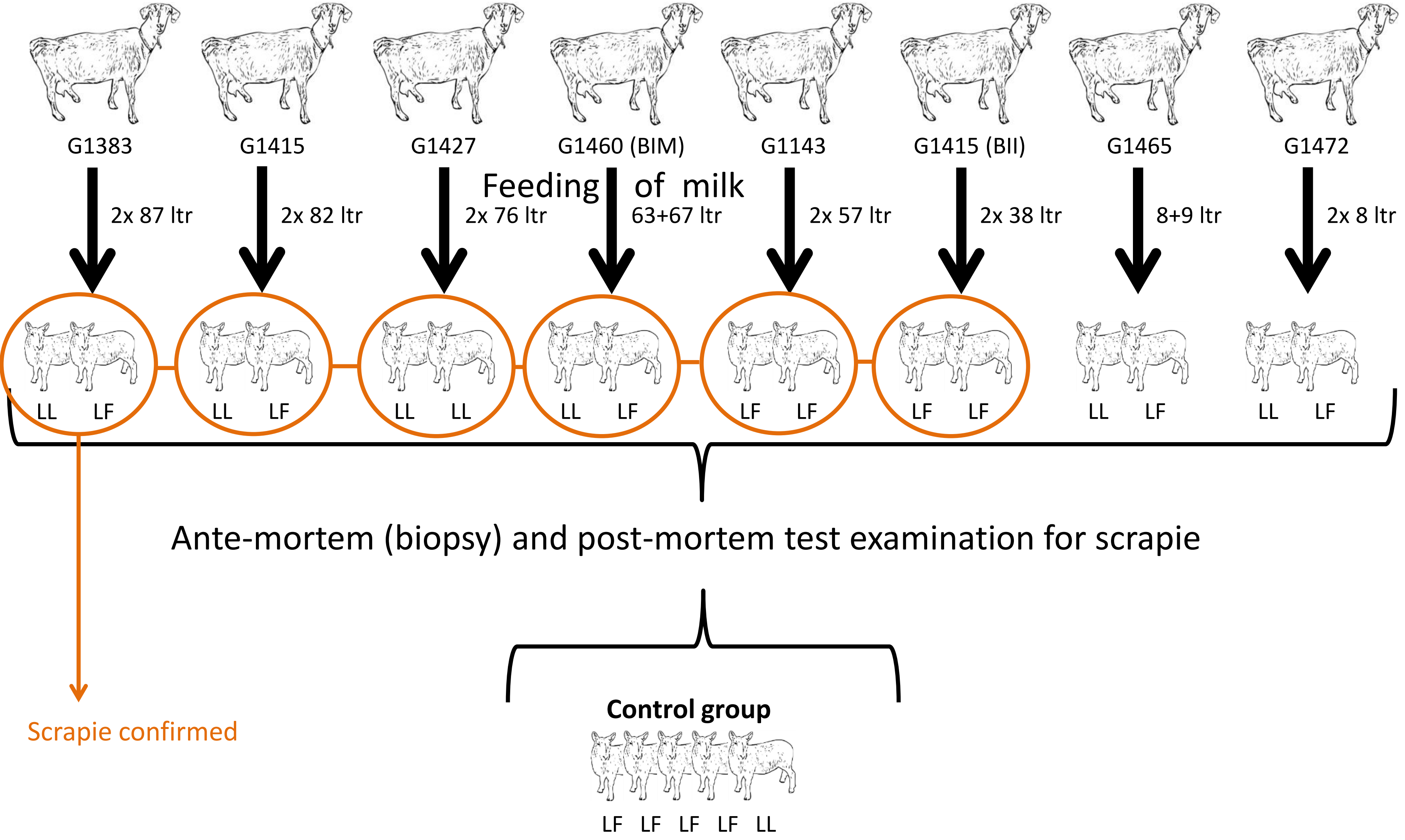

Supplement: Additional file 1: — Schematic summary of the study. This file provides a graphical overview of the design of the pilot and milk transmission study and the overall results. (PDF 301 kb) [file 12917_2016_807_MOESM1_ESM.pdf]
